# Food Adulteration

**Published:** 1883-05

**Authors:** 


### FOOD ADULTERATION.

The evils resulting from the use of Adulterated Baking Powders culminated a few years ago in a popular war against the products of manufacturers who substituted Alum for Cream of Tartar.

Whenever public feeling becomes aroused against an evil there is usually a strong effort made to abate it ; and so, in this instance, almost the entire community seemed to protest against being longer fed on a questionable article that found its way into "the staff of life ;" but it seems impossible to wholly destroy a business conducted by heartless men, and that affords enormous profits. The only way is to warn people against the use of adulterated and poisonous preparations of every sort, and make our warning effective by stating what preparations are pure and reliable. This is a matter within the province of the JOURNAL OF HEALTH, and an honest statement of the facts, without fear or favor, is due to its readers. We say then : as you value your health, and the health of those under your care, avoid the use of all foods, candies, baking powders, and other articles, whose purity is questionable.

There is also a choice in the products put into the market by reputable manufacturers.

The best baking powder is made from pure Cream of Tartar, Bicarbonate of Soda, and a small quantity of flour or starch. Frequently other ingredients are used, and serve a purpose in reducing the cost and increasing the profits of the manufacturer.

We give the Government Chemist's analyses of two of the leading baking powders :

> I have examined samples of "Cleveland's Superior Baking Powder" and "Royal Baking Powder," purchased by myself in this city, and I find they contain :
>
> **"Cleveland's Superior Baking Powder."**
>
> Cream of Tartar.
> Bicarbonate of Soda.
> Flour.
>
> Available carbonic acid gas 12.61 per cent., equivalent to 118.2 cubic inches of gas per oz. of Powder.
>
> ---
>
> **"Royal Baking Powder."**
>
> Cream of Tartar.
> Bicarbonate of Soda.
> Carbonate of Ammonia.
> Tartaric Acid.
> Starch.
>
> Available carbonic acid gas 12.40 per cent., equivalent to 116.2 cubic inches of gas per oz. of Powder.
>
> Ammonia gas 0.43 per cent., equivalent to 10.4 cubic inches per oz. of Powder.
>
> Note.—The Tartaric Acid was doubtless introduced as free acid, but subsequently combined with ammonia, and exists in the Powder as a Tartrate of Ammonia. E. G. LOVE, Ph.D.
>
> NEW YORK, Jan. 17th, 1881.

The above analyses indicate a preference for "Cleveland's Superior Baking Powder," and our opinion is that it is the better preparation.

